# Retraction: Extreme Resistance as a Host Counter-counter Defense against Viral Suppression of RNA Silencing

**DOI:** 10.1371/journal.ppat.1005207

**Published:** 2015-09-22

**Authors:** Raphaël Sansregret, Vanessa Dufour, Mathieu Langlois, Fouad Daayf, Patrice Dunoyer, Olivier Voinnet, Kamal Bouarab

At the request of the authors, *PLOS Pathogens* is retracting this publication following an investigation into concerns about the origin and assembly of Figure 6 and a mounting mistake in Figure 1B.

The Northern blot depicted in Figure 6 contains several band duplications affecting the panels labelled 'IP@HA' and 'total RNA'. The figure was provided by Patrice Dunoyer during revision and was extracted from the Master thesis of a former student working under his supervision, without the prior consultation or consent of this student. The other authors of Sansregret *et al*. were not informed about the origin of this figure and, regrettably, its erroneous content escaped their attention both at the final revision and proofreading stages.

During inspection of the original blots, we realised that the loading control of Figure 1B was not the correct one; we have found the cognate one and the loadings are comparable. Importantly, the cross-reacting band visible on Figure 1B also provides an internal loading control. This mistake was made by co-authors Raphael Sansregret and Kamal Bouarab.

Further analysis of the raw material underlying the results depicted in Figure 6 was found to support the original conclusions drawn from it. The other conclusions of the published article also remain valid. However, given the nature and extent of data manipulation in Figure 6, the authors have collectively decided to retract the study.

All authors concur with this statement and apologise for not having detected these errors. Kamal Bouarab and Olivier Voinnet, as the corresponding authors, take full responsibility for the publication of this erroneous paper and regret deeply the inconvenience caused.
